# The relationship between *Helicobacter pylori* severity and hematological parameters in pediatric patients with celiac disease

**DOI:** 10.1186/s12887-026-06694-w

**Published:** 2026-03-09

**Authors:** Bilal Arslan, Adnan Erseçkin, Burhan Beger, Eyyüp Yürektürk, Serap Karaman, Burcu Güven, Orhan Beger, Mecnun Çetin

**Affiliations:** 1 Department of Pediatrics, Van Yüzüncü Yıl University Dursun Odabaş Medical Center, Van, 65080 Türkiye Turkey; 2 Department of Pediatric Surgery, Van Yüzüncü Yıl University Faculty of Medicine, Van, Türkiye Turkey; 3 Department of Neonatology, Van Yüzüncü Yıl University Dursun Odabaş Medical Center, Van, Türkiye Turkey; 4 Department of Pediatric Gastroenterology, Faculty of Medicine, Karadeniz Technical University, Trabzon, Türkiye Turkey; 5 Department of Anatomy, Gaziantep University Faculty of Medicine, Gaziantep, Türkiye Turkey; 6 Department of Pediatric Cardiology, Private Practice Clinic, Van, Türkiye Turkey

**Keywords:** Celiac disease, *Helicobacter pylori*, Hemoglobin, Anemia, Pediatric gastroenterology, Histopathology, Immunomodulator

## Abstract

**Aim:**

The relationship between *Helicobacter pylori* infection and hematological parameters in pediatric celiac disease remains controversial. While *H. pylori* has traditionally been associated with iron deficiency, emerging data suggest a more complex interaction.

**Materials and methods:**

This retrospective observational study included 103 pediatric patients with biopsy-confirmed celiac disease diagnosed between 2010 and 2024. H. pylori status and severity were determined histopathologically using gastric biopsies. Hematological and biochemical parameters obtained at diagnosis were compared across *H. pylori* severity groups. Group comparisons were performed using Kruskal–Wallis and Mann–Whitney U tests with Bonferroni correction. Multinomial logistic regression analysis was conducted to evaluate factors associated with increasing *H. pylori* severity.

**Results:**

Hemoglobin and hematocrit levels differed significantly across *H. pylori* severity groups in univariate analyses (*p* < 0.05). Post-hoc analyses demonstrated significantly higher hemoglobin and hematocrit levels in the 2 + *H. pylori*–positive group compared with the 1 + group after Bonferroni correction. In addition, significantly higher Hb level and Htc level were found in 3 + *H. pylori*–positive group compared with 1 + *H. pylori*–positive group. No significant differences were observed in ferritin or mean corpuscular volume levels. In multivariable analyses, *H. pylori* severity was not identified as an independent predictor of hematological parameters.

**Conclusion:**

In pediatric patients with newly diagnosed celiac disease, *H. pylori* positivity was associated with modest differences in hemoglobin and hematocrit levels in univariate analyses; however, these associations did not persist after adjustment for potential confounders. These findings suggest that *H. pylori* infection does not independently influence hematological status in this population. Further prospective studies incorporating direct immunological and iron metabolism markers are warranted to clarify underlying mechanisms.

## Introduction

Celiac disease is an immune-mediated enteropathy characterized by villous damage in the small intestine following gluten ingestion in genetically predisposed individuals. The disease may present with gastrointestinal symptoms, growth retardation, iron deficiency anemia, and various extraintestinal findings [1, 2]. Iron deficiency and related hematological disorders are among the most common and earliest complications in celiac patients, resulting from intestinal villous damage and impaired iron absorption [3].

*Helicobacter pylori (H. pylori),* a Gram-negative microorganism infecting approximately half of the world’s population, is associated with gastritis, peptic ulcers, and gastric cancer [4]. The impact of *H. pylori* on iron metabolism, however, is more complex. While some studies indicate that chronic gastritis reduces iron absorption and leads to iron deficiency [5, 6], others suggest that this relationship may be weaker and more variable in the pediatric population [7].

Recent studies have examined the relationship between celiac disease and *H. pylori*. Although several reports suggest that *H. pylori *positivity increases the risk of anemia in celiac patients [8, 9], some studies indicate that the coexistence of these two conditions may unexpectedly improve hematological parameters [10]. Due to its immune-modulating properties, whether *H. pylori* plays a silent regulatory role in the systemic inflammation and malabsorption associated with celiac disease has been a subject of debate in recent years [11, 12].

Large meta-analyses published in 2021 and 2022 showed that the prevalence of *H. pylori* in celiac patients may be lower than in the general population and that this relationship varies according to geographical region, diagnostic methodology, and bacterial strain differences [13, 14]. Additionally, strains possessing virulence factors such as CagA and VacA have been shown to significantly alter disease severity and immune responses [15]. A recent study from Turkey also emphasized that the prevalence of *H. pylori* infection in celiac patients is low and that the underlying mechanism remains unclear [16].

In this context, the unexpected increase in hemoglobin (Hb) and hematocrit (Htc) levels associated with *H. pylori* positivity in celiac patients is a striking clinical observation that challenges the classical understanding of the coexistence of the two diseases. Current literature indicates that the effects of *H. pylori* on iron metabolism are not unidirectional and may vary depending on host immune responses and environmental factors [12, 17].

In our study, we aimed to investigate the effects of *H. pylori* positivity on hematological parameters in celiac patients, to evaluate the clinical implications of the coexistence of these two diseases, and to shed light on the potential silent modulatory role of *H. pylori* infection.

## Materials and methods

### Study design and patient selection

This study is an observational study retrospectively examining data from patients diagnosed with celiac disease in a pediatric gastroenterology clinic between 2010 and 2024. All patients underwent upper gastrointestinal endoscopy during the diagnostic process, and biopsy samples were taken from the duodenum to determine the diagnosis of celiac disease.

During upper gastrointestinal endoscopy, gastric and duodenal biopsy specimens were obtained in all patients as part of routine clinical practice. According to institutional practice, at least one antral biopsy and multiple duodenal biopsy samples (typically ranging from two to four per procedure) were collected during each endoscopic examination. Due to the retrospective design of the study, the exact anatomical localization of duodenal biopsies (duodenal bulb versus distal duodenum) was not consistently specified in the archived pathology reports. However, all available specimens were histopathologically adequate for evaluation of villus–crypt architecture and intraepithelial lymphocyte density. Duodenal biopsies were graded according to the Marsh–Oberhuber classification, and gastric biopsies were assessed using the Updated Sydney System for *Helicobacter pylori* density and inflammatory activity.

### Histopathological evaluation and diagnostic criteria

Celiac disease diagnosis and mucosal damage grading were performed according to the Marsh–Oberhuber classification. Duodenal biopsies and gastric antrum and corpus biopsies were evaluated independently (blindly) from clinical and laboratory data by two pathologists experienced in celiac disease and *Helicobacter pylori* histopathology. In case of discrepancies between the evaluations, the consensus prevailed.

Duodenal biopsies were prepared and examined in a manner suitable for histopathological examination, allowing for the assessment of villus-crypt architecture and intraepithelial lymphocyte proliferation.

### Anthropometric measurements

At the time of diagnosis, patients’ height, weight, and body mass index (BMI) measurements were recorded. World Health Organization (WHO) percentile and Z-score references were used for height–weight and BMI assessments. Malnutrition was classified according to WHO Z-score criteria.

### Biochemical and hematological parameters

Complete blood count, iron parameters, ferritin level, and routine biochemical test results obtained at the time of diagnosis were recorded. Hemoglobin (Hb), hematocrit (Htc), mean erythrocyte volume (MCV), and ferritin values ​​were used as the primary hematological variables in the analyses. Age and gender-appropriate reference ranges were used in the evaluation of laboratory parameters.

### H. pylori evaluation

*H. pylori* infection was evaluated by histopathological examination of antrum and corpus biopsies obtained during upper gastrointestinal endoscopy. All samples were examined with hematoxylin-eosin (H&E) and, if necessary, Giemsa stain. The Updated Sydney System was used for gastrointestinal pathology assessment. Infection severity was classified as:

- Negative
- Positive 1+ (mild)
- Positive 2+ (moderate)
- Positive 3+ (severe)

Histopathological evaluations were performed blindly by two pathologists, and consensus was reached in case of disagreement. Due to the retrospective nature of the study, additional diagnostic methods such as rapid urease test, stool antigen test, or urea breath test were not used.

### Missing data management

Patient records were screened for missing data prior to analysis. Cases with more than 10% missing data in specific variables were excluded from the analysis during the preliminary evaluation phase. Following this preliminary screening, 103 patients were included in the study whose clinical, anthropometric, and laboratory data were complete, and the final analysis was performed on the patient group without missing data. The patient selection and exclusion process is shown in the patient flowchart.

### Statistical analysis

Statistical analyses were performed using SPSS (IBM, Armonk, NY). Data were checked for normal distribution using the Shapiro-Wilk test. Changes in data by gender were analyzed using the Mann-Whitney U test. Changes in parameters according to diagnosis and *H. pylori *severity were analyzed using the Kruskal-Wallis test. After significance was found in the Kruskal–Wallis test, pairwise comparisons between groups were performed using the Mann–Whitney U test, and Bonferroni correction was applied. The distribution of patients in percentile groups according to the first and last measurements was examined using the chi-square test. The relationship between the presence or absence of *H. pylori* and the diagnosis type was examined using the chi-square test. To evaluate factors associated with increasing *H. pylori* severity (negative, + 1, +2, and + 3), multinomial logistic regression analysis was performed, using *H. pylori* negative status as the reference category. Age, Hb, Htc, and ferritin were included as continuous covariates. Sex and Marsh classification were included as categorical variables, with female sex and Marsh type 1 defined as reference categories. Given the limited number of patients in higher *H. pylori* severity categories, a reduced multinomial model focusing on clinically relevant variables was constructed to minimize model overfitting. Marsh classification was evaluated using a five-category model (type 1, type 2, type 3a, type 3b, and type 3c). Odds ratios (ORs) with 95% confidence intervals (CIs) were calculated. Ferritin levels were scaled per 100 ng/mL to improve interpretability of the regression coefficients. All statistical tests were two-sided, and a *p* < 0.05 was considered statistically significant.

## Results

The study population comprised 103 pediatric patients, including 45 females (8.40 ± 4.06 years, range: 1–16 years) and 58 males (8.59 ± 4.16 years, range: 1–15 years). The overall mean age was 8.50 ± 4.10 years (range: 1–16 years).

Changes in anthropometric and laboratory parameters according to *H. pylori* severity were shown in Table 1. Hb level (Kruskal–Wallis H = 9.02, *p* = 0.029) and Htc level (Kruskal–Wallis H = 9.75, *p* = 0.021) differed significantly among the four groups. Following significant Kruskal–Wallis tests for Hb and Htc levels across H. pylori severity groups, pairwise comparisons were performed using the Mann–Whitney U test with Bonferroni correction (adjusted significance threshold *p* < 0.008). Post-hoc pairwise comparisons showed significantly higher Hb level (*p* = 0.005, *r* = 0.51) and Htc level (*p* = 0.002, *r* = 0.54) in 2 + *H. pylori*–positive group compared with 1 + *H. pylori*–positive group. In addition, we found significantly higher Hb level (*p* = 0.033, *r* = 0.40) and Htc level (*p* = 0.021, *r* = 0.42) in 3 + *H. pylori*–positive group compared with 1 + *H. pylori*–positive group. All remaining pairwise comparisons for Hb and Htc were not statistically significant after correction.

**Table 1:** Comparison of parameters in terms of *H. pylori* severity (Kruskal-Wallis test)

Parameters	Negative (*n* = 58)	Positive (+ 1) (*n* = 19)	Positive (+ 2) (*n* = 11)	Positive (+ 3) (*n* = 15)	*p* value
Age (years)	8 (1–16)	7 (1–15)	9 (5–15)	12 (4–15)	0.055
Height (cm)	117 (71–173)	117 (72–161)	124 (101–151)	137 (101–156)	0.166
Weight (kg)	22 (7.60–55)	21 (8.60–55)	22 (17–45)	26 (14–44)	0.198
Hb (g/dL)	12.60 (5.30–14.90)	11.30 (7.40–15.40)	13.40 (11-16.10)	13.30 (8.40–14.90)	**0.029**
Htc (%)	37.35 (17.90–44.60)	34 (24.50–44)	39 (36–50)	39 (28.90–46)	**0.021**
MCV (fL)	77.50 (54-89.20)	75.20 (54.70–89.60)	81 (75–89)	78 (55-87.50)	0.056
WBC (×10⁹/L)	8,200 (3,600 − 17,800)	8,100 (4,900 − 18,600)	7,500 (4,300 − 13,400)	7,900 (4,100 − 12,600)	0.879
ANC (×10⁹/L)	3,900 (1,000–9,400)	4,000 (1,600 − 14,200)	4,200 (1,400 − 11,600)	4,200 (1,500-8,500)	0.980
ALC (×10⁹/L)	3,050 (1,100-7,000)	3,200 (1,500-4,600)	2,300 (800-3,670)	2,600 (1,400-4,000)	0.103
PLT (×10⁹/L)	345,000 (170,000-952,000)	340,000 (234,000-900,000)	312,000 (244,000- 429,000)	349,000 (257,000-839,000)	0.858
B12 (pg/mL)*	292 (140–946)	290 (180–768)	333 (182–728)	314 (153–737)	0.602
Ferritin (ng/mL)*	20 (1-121)	15 (1–81)	25 (9–38)	8 (1–52)	0.420
Vitamin D (ng/mL)*	20 (5–67)	24 (9.40–40)	18 (7.50–37)	19 (12–22)	0.689
Folate (ng/mL)	7.15 (1–21)	5.70 (2-14.40)	7.30 (4.70–11.10)	4.40 (1.80–13.50)	0.735

Data for these parameters were not available for some patients. Because the data were not normally distributed, they are presented as median (minimum-maximum) in the table. Bold values ​​indicate parameters that are statistically significant. *Hb* Hemoglobin, *Htc* Hematocrit, *MCV* Mean Corpuscular Volume, *WBC* White Blood Cell count, *ANC* Absolute Neutrophil Count, *ALC* Absolute Lymphocyte Count, *PLT* Platelet count

A reduced multinomial logistic regression analysis was performed to evaluate factors associated with increasing *H. pylori* severity, using *H. pylori*–negative patients as the reference category (Table 2). Age, hemoglobin, hematocrit, ferritin, sex, and Marsh classification (five-category model) were included in the analysis. Across all *H. pylori* severity categories (+ 1, + 2, and + 3), no variable demonstrated an independent statistically significant association after multivariable adjustment (all *p* > 0.05). Age and Hb levels were not associated with *H. pylori* severity at any level. Htc showed an inverse trend with increasing *H. pylori* severity; however, this association did not reach statistical significance. Similarly, ferritin levels demonstrated modest increases in odds ratios with higher *H. pylori* grades, but these findings were not statistically significant. Sex was not independently associated with *H. pylori* severity. Although male sex tended to show higher odds ratios in higher severity categories, the confidence intervals were wide and included unity. Regarding histopathological severity, higher Marsh subtypes (types 3a, 3b, and 3c) exhibited progressively increasing odds ratios for more severe *H. pylori* infection. Nevertheless, these associations were accompanied by wide confidence intervals and did not reach statistical significance, likely reflecting limited statistical power due to small subgroup sizes. Overall, the multivariable analysis indicated that none of the evaluated demographic, hematological, or histopathological parameters independently predicted *H. pylori* severity in this cohort.

**Table 2:** Factors associated with *H. pylori* severity (Reduced multinomial logistic regression analysis)

Variable	H. pylori + 1 OR (95% CI)	*p* value	H. pylori + 2 OR (95% CI)	*p* value	H. pylori + 3 OR (95% CI)	*p* value
Age (years)	1.04 (0.95–1.14)	0.360	1.07 (0.96–1.19)	0.220	1.09 (0.94–1.26)	0.250
Hb (g/dL)	0.89 (0.66–1.19)	0.430	0.84 (0.60–1.17)	0.300	0.78 (0.52–1.16)	0.220
Htc (%)	0.97 (0.90–1.05)	0.480	0.95 (0.87–1.04)	0.290	0.93 (0.83–1.05)	0.260
Ferritin (per 100 ng/mL)	1.08 (0.86–1.35)	0.520	1.12 (0.88–1.44)	0.350	1.18 (0.89–1.56)	0.250
Male sex (ref: female)	1.21 (0.46–3.18)	0.700	1.34 (0.47–3.85)	0.580	1.49 (0.41–5.39)	0.540
Marsh type 2 (ref: type 1)	1.15 (0.36–3.62)	0.810	1.38 (0.41–4.61)	0.590	1.52 (0.35–6.63)	0.570
Marsh type 3 A (ref: type 1)	1.29 (0.42–3.96)	0.660	1.61 (0.50–5.18)	0.430	1.84 (0.44–7.71)	0.410
Marsh type 3B (ref: type 1)	1.41 (0.39–5.02)	0.600	1.88 (0.53–6.70)	0.330	2.12 (0.48–9.41)	0.320
Marsh type 3 C (ref: type 1)	1.58 (0.41–6.04)	0.510	2.07 (0.56–7.66)	0.270	2.48 (0.53–11.53)	0.250

Outcome reference category: *H. pylori* negative. Predictor reference categories: Female sex; Marsh type 1. Abbreviations: *OR* odds ratio, *CI* confidence interval. A reduced multinomial logistic regression model including age, sex, hemoglobin, hematocrit, ferritin, and Marsh classification (five-category model) was constructed to minimize model overfitting due to the limited number of patients in higher *H. pylori* severity categories. Odds ratios (ORs) with 95% confidence intervals (CIs) are presented. Ferritin was scaled per 100 ng/mL for interpretability. No Marsh subtype showed an independent association with increasing *H. pylori* severity after adjustment. *Hb* Hemoglobin, *Htc* Hematocrit

The comparison of parameters according to Marsh subtypes was presented in Table 3. No statistically significant differences were found among the categories.

**Table 3:** Comparison of parameters according to Marsh subtypes (Kruskal-Wallis test)

Parameters	Type 1 (*n* = 7)	Type 2 (*n* = 4)	Type 3 A (*n* = 14)	Type 3B (*n* = 40)	Type 3 C (*n* = 38)	*p*
Age	10 (4–15)	11 (3–15)	7 (1–15)	9 (1–15)	10 (1–16)	0.280
Height	125 (90–156)	134 (93–151)	108 (72–163)	124.50 (71–161)	123 (72–173)	0.499
Weight	23 (14–40)	29 (13–45)	17.50 (7.60–55)	23 (8.50–55)	22.75 (8.60–50)	0.559
Hb	12.90 (5.30–14.40)	12.85 (12.40–16.10)	12.50 (9.70–14.90)	13.25 (9.10–15.40)	12.05 (7.40–14.90)	0.070
Htc	39.10 (17.90–42.20)	37.70 (35–50)	37.85 (27–44)	38 (29-44.60)	36 (24.50–46)	0.540
MCV	76 (54-86.10)	81 (78–89)	76.70 (65-87.50)	79.50 (63-89.60)	75.60 (54.70–89.20)	0.057
WBC	9,100 (7,200 − 14,100)	7,800 (5,400-8,500)	8,800 (5,000–17,800)	8,450 (4,100 − 18,600)	7,750 (3,600 − 17,500)	0.488
ANC	5,400 (3,800-7,900)	3,700 (2,400-4,000)	4,550 (2,600-7,900)	3,800 (1,000–11,600)	3,850 (1,400 − 14,200)	0.180
ALC	2,400 (1,100-4,600)	2,800 (2,300-3,600)	3,200 (1,800-7,000)	2,950 (800-6,700)	2,600 (1,400-4,600)	0.461
PLT	349,000 (298,000-952,000)	367,500 (234,000-455,000)	345,000 (213,000-604,000)	330,500 (218,000-582,000)	351,000 (170,000-900,000)	0.450
B12*	462 (233–946)	387 (305–728)	323.50 (140–901)	279 (150–880)	292 (146–768)	0.092
Ferritin*	12 (1–55)	26 (19–38)	29.50 (3–80)	21 (3-121)	11 (1–57)	0.057
Vitamin D*	25 (10–32)	8 (7.50–13)	23 (13–40)	18 (5–67)	20 (8–38)	0.069
Folate*	5.90 (1.30–10.90)	6.85 (4.70-9)	6.40 (2.20–21)	7.30 (2.20–20.50)	6.20 (1-14.40)	0.995

Data for these parameters were not available for some patients. Because the data were not normally distributed, they are presented as median (minimum-maximum) in the table. *Hb* Hemoglobin (g/dL), *Htc* Hematocrit (%), *MCV* Mean Corpuscular Volume (fL), *WBC* White Blood Cell count (×10⁹/L), *ANC* Absolute Neutrophil Count (×10⁹/L), *ALC *Absolute Lymphocyte Count (×10⁹/L), *PLT* Platelet count (×10⁹/L), B12: Vitamin B12 (pg/mL), Folate: ng/mL, Ferritin: ng/mL, Vitamin D: ng/mL, Age: years, Height: cm, Weight: kg

Fifteen of the 103 patients discontinued treatment. The remaining 88 patients were re-evaluated after one year with repeat height and weight measurements. Among those who were prescribed a dietary program, 76 adhered to the diet, while 12 did not. At one year, the median height and weight of the 88 patients were 128 cm (78–173 cm) and 26.5 kg (10–64 kg), respectively. Compared with baseline measurements (height: 122 cm; weight: 22.5 kg), both height and weight increased significantly (*p* < 0.001).

Among patients who adhered to the dietary program, both height (121.5 cm vs. 128 cm) and weight (22 kg vs. 27 kg) increased significantly over one year (*p* < 0.001 for both). In patients who did not adhere to the diet, height increased from 111 cm to 114.5 cm (*p* = 0.003) and weight from 17.5 kg to 19 kg (*p* = 0.027). However, there were no statistically significant differences between diet-adherent and non-adherent patients in terms of final height (*p* = 0.143) or weight (*p* = 0.088).

The distribution of patients according to percentile categories based on baseline and one-year height and weight measurements was shown in Table 4. No statistically significant changes were observed in percentile distribution after one year.

**Table 4:** Distribution of patients in percentile groups according to the first and last measurements (Chi-square test)

Percentile Groups	Height		Weight	
	First	Last	First	Last
< 3	49 (47.6%)	32 (36.4%)	43 (41.7%)	27 (30.7%)
3–10	20 (19.4%)	13 (14.8%)	23 (22.3%)	12 (13.6%)
10–25	16 (15.4%)	16 (18.2%)	17 (16.5%)	20 (22.7%)
25–50	12 (11.7%)	13 (14.8%)	11 (10.7%)	17 (19.3%)
50–75	4 (3.9%)	11 (12.5%)	7 (6.8%)	10 (11.4%)
75–90	1 (1%)	2 (2.2%)	2 (2%)	2 (2.3%)
> 90	1 (1%)	1 (1.1%)	-	-
p	0.272		0.154	

Last measurements were available for 88 patients who completed the one-year follow-up

The distribution of diagnostic categories according to *H. pylori* status was presented in Table 5. There was no statistically significant association between *H. pylori* positivity and Marsh subtypes (*p* = 0.062). Therefore, histopathological examination revealed no significant association between Marsh classification and *H. pylori* positivity. The degree of villous atrophy, crypt hyperplasia, and intraepithelial lymphocyte distribution were similar between the groups.

**Table 5:** Relationship between the presence or absence of *H. pylori* and Marsh subtypes (Chi-square test)

Types	Negative	Positive	Total	*p*
Type 1	6 (10.3%)	1 (2.2%)	7 (6.8%)	0.062
Type 2	1 (1.7%)	3 (6.7%)	4 (3.9%)	
Type 3 A	11 (19%)	3 (6.7%)	14 (13.6%)	
Type 3B	23 (39.7%)	17 (37.8%)	40 (38.8%)	
Type 3 C	17 (29.3%)	21 (46.7%)	38 (36.9%)	
total	58 (56.3%)	45 (43.7%)	103	

Similarly, no significant association was found between *H. pylori* status and pre- or post-treatment percentile categories (Table 6). In other words, *H. pylori* infection and its treatment were not associated with a statistically significant change in growth percentiles.

**Table 6:** Relationship between the presence or absence of *H. pylori* and percentile groups (Chi-square test)

Percentile groups	First height		First weight		Last height		Last weight	
	Negative	Positive	Negative	Positive	Negative	Positive	Negative	Positive
< 3	24 (41.5%)	25 (55.6%)	19 (32.8%)	24 (53.3%)	15 (30.6%)	17 (43.6%)	12 (24.5%)	15 (38.5%)
3–10	12 (20.7%)	8 (17.8%)	14 (24.2%)	9 (20%)	6 (12.2%)	7 (17.9%)	7 (14.3%)	5 (12.8%)
10–25	10 (17.2%)	6 (13.3%)	10 (17.2%)	7 (15.6%)	11 (22.4%)	5 (12.9%)	13 (26.5%)	7 (17.9%)
25–50	8 (13.8%)	4 (8.9%)	8 (13.8%)	3 (6.7%)	6 (12.2%)	7 (17.9%)	11 (22.4%)	6 (15.4%)
50–75	2 (3.4%)	2 (4.4%)	5 (8.6%)	2 (4.4%)	8 (16.3%)	3 (7.7%)	4 (8.2%)	6 (15.4%)
75–90	1 (1.7%)	0	2 (3.4%)	0	2 (4.2%)	0	2 (4.1%)	0
> 90	1 (1.7%)	0	0	0	1 (2.1%)	0	0	0
p	0.735		0.286		0.344		0.384	

## Discussion

This study evaluated the relationship between the severity of *Helicobacter pylori* infection and hematological, anthropometric, and histopathological parameters in pediatric patients diagnosed with biopsy-confirmed celiac disease. Our findings show statistically significant differences in hemoglobin and hematocrit levels in univariate analyses based on *H. pylori* severity; however, this relationship was not confirmed as an independent predictor in multivariate analyses.

### Interpretation of hemoglobin and hematocrit findings

The main pathophysiological mechanism of anemia in celiac disease is impaired iron absorption due to villous atrophy developing in the proximal small intestine [18]. However, in our study, comparisons made according to the severity of *H. pylori* infection revealed that hemoglobin and hematocrit levels were higher in the 2 + and 3 +* H. pylori* positive groups compared to the 1 + positive group. This finding suggests that *H. pylori* infection may be associated with hematological parameters in celiac patients. However, these differences lost their statistical significance in multivariate analyses performed by correcting for potential confounding variables such as age, gender, ferritin level, and histopathological disease severity. This suggests that the observed Hb and Htc differences may be the result of a multifactorial interaction rather than an independent effect specific to *H. pylori* infection. The literature also reports inconsistent results regarding the relationship between *H. pylori* infection and hemoglobin levels, especially in the pediatric population [19, 20].

### Evaluation of ferritin and MCV findings

In our study, no statistically significant difference was found in ferritin and MCV levels according to *H. pylori* severity. This finding suggests that the limited differences observed in hemoglobin and hematocrit do not reflect a true improvement in iron stores. It is known that ferritin can act as an acute phase reactant in the presence of inflammation and therefore may not always accurately reflect iron stores [19, 21]. This suggests that the *H. pylori*-related inflammatory response may indirectly affect hemoglobin levels through hepcidin-mediated erythropoiesis regulation, but may not lead to a true increase in iron stores. In this context, it can be said that the effect of *H. pylori* infection on hematological parameters in celiac patients is limited and indirect.

### Histopathological findings and the relationship with *H. pylori*

In our study, no statistically significant relationship was found between the severity of histopathological disease, assessed according to the Marsh–Oberhuber classification, and *H. pylori* positivity or infection severity. The fact that villous atrophy, crypt hyperplasia, and intraepithelial lymphocyte distribution are independent of *H. pylori* status suggests that *H. pylori* infection is not a key factor determining the degree of mucosal damage in celiac disease. This finding is consistent with previous studies reporting an inverse or weak association between *H. pylori* infection and celiac disease [10, 14].

### Anthropometric findings and growth parameters

In the one-year follow-up, it was observed that the increases in height and weight were mainly related to adherence to the gluten-free diet, and *H. pylori* infection did not have a significant effect on growth parameters. The fact that no significant difference was found in final height and weight between patients who adhered to the diet and those who did not supports the idea that *H. pylori* infection does not play a decisive role in growth. These findings are consistent with previous pediatric studies reporting that the main determinant of growth and development in celiac patients is dietary treatment [22].

### Limited interpretation of the immunomodulation hypothesis

Some studies have suggested that *H. pylori* infection may affect the course of autoimmune diseases by modulating the immune response [23]. However, cytokine profiles, T-regulatory cell response, or other immunological parameters were not directly evaluated in this study. Therefore, comments regarding the possible immunomodulatory effects of *H. pylori* infection in celiac disease should only be considered at the hypothesis level and should not be interpreted as a causal relationship.

### Overall assessment

In conclusion, this study found significant associations between *H. pylori* infection severity and hemoglobin and hematocrit levels in univariate analyses; however, these associations were not confirmed as independent predictors in multivariate analyses. The findings suggest that *H. pylori *infection may have a limited and indirect effect on hematological parameters in celiac patients, but do not support a strong causal relationship from a clinical perspective, and suggest that further studies are needed in this regard.

## Conclusion

In conclusion, significant associations were found between the severity of *H. pylori* infection and hemoglobin and hematocrit levels in univariate analyses in pediatric cases with biopsy-confirmed celiac disease; however, these associations were not confirmed as independent predictors in multivariate analyses adjusted for age, sex, ferritin level, and histopathological severity. Therefore, our findings suggest that *H. pylori* may have a limited and indirect effect on hematological parameters, but do not support a causal relationship and point to the need for larger, prospective studies.

### Study limitations

This study has several limitations. Firstly, the retrospective and single-center design of the study makes it difficult to establish a causal relationship between the findings and limits their generalizability. Secondly, *Helicobacter pylori* infection was evaluated only by histopathological examination, and additional diagnostic methods such as rapid urease test, stool antigen test, or urea breath test were not used. This may have led to the classification of infection severity as lower or higher than it actually is, especially in cases showing patchy colonization.

Thirdly, the limited number of patients in higher *H. pylori* severity categories (+ 2 and + 3) may have reduced the statistical power in multivariate analyses and may have led to some relationships not reaching statistical significance. In addition, ferritin level alone was used to evaluate iron metabolism; additional biochemical and immunological parameters such as hepcidin, transferrin saturation, soluble transferrin receptor, and inflammatory markers could not be evaluated. This situation limits the detailed elucidation of the mechanisms underlying the hematological effects of *H. pylori* infection.

Finally, although anthropometric assessments were performed during the one-year follow-up, the relatively small number of patients who did not adhere to their diet and the effect of age-related physiological increase on growth may have made it difficult to determine possible relationships between *H. pylori* infection and growth parameters. Considering these limitations, the findings should be interpreted cautiously and supported by prospective, multicenter studies.

## Data Availability

The datasets generated and/or analyzed during the current study are not publicly available due to patient confidentiality but are available from the corresponding author on reasonable request and with institutional permission.

## Abbreviations

Hb: Hemoglobin
Htc: Hematocrit
MCV: Mean Corpuscular Volume
WBC: White Blood Cell
PLT: Platelet
BMI: Body Mass Index
WHO: World Health Organization
